# Chronic Cough-Related Differences in Brain Morphometry in Adults: A Population-Based Study

**DOI:** 10.1016/j.chest.2023.02.007

**Published:** 2023-02-11

**Authors:** Johnmary T. Arinze, Elisabeth J. Vinke, Katia M.C. Verhamme, Maria A.J. de Ridder, Bruno Stricker, M.K. Ikram, Guy Brusselle, Meike W. Vernooij

**Affiliations:** a Department of Respiratory Medicine, Ghent University Hospital, Ghent, Belgium; b Department of Medical Informatics, Erasmus Medical Center, Rotterdam, The Netherlands; c Department of Epidemiology, Erasmus Medical Center, Rotterdam, The Netherlands; d Department of Radiology and Nuclear Medicine, Erasmus Medical Center, Rotterdam, The Netherlands; e Department of Respiratory Medicine, Erasmus Medical Center, Rotterdam, The Netherlands

**Keywords:** adults, brain MRI, brain volume analysis, chronic cough, intracranial volume, population-based study

## Abstract

**Background:**

Individuals with cough hypersensitivity have increased central neural responses to tussive stimuli, which may result in maladaptive morphometric changes in the central cough processing systems.

**Research Question:**

Are the volumes of the brain regions implicated in cough hypersensitivity different in adults with chronic cough compared with adults without chronic cough?

**Study Design and Methods:**

Between 2009 and 2014, participants in the Rotterdam Study, a population-based cohort, underwent brain MRI and were interviewed for chronic cough, which was defined as daily coughing for at least 3 months. Regional brain volumes were quantified with the use of parcellation software. Based on literature review, we identified and studied seven brain regions that previously had been associated with altered functional brain activity in chronic cough. The relationship between chronic cough and regional brain volumes was investigated with the use of multivariable regression models.

**Results:**

Chronic cough was prevalent in 9.6% (No. = 349) of the 3,620 study participants (mean age, 68.5 ± 9.0 years; 54.6% female). Participants with chronic cough had significantly smaller anterior cingulate cortex volume than participants without chronic cough (mean difference, ˗126.16 mm^3^; 95% CI, −245.67 to −6.66; *P* = .039). Except for anterior cingulate cortex, there were no significant difference in the volume of other brain regions based on chronic cough status. The volume difference in the anterior cingulate cortex was more pronounced in the left hemisphere (mean difference, −88.11 mm^3^; 95% CI, −165.16 to −11.06; *P* = .025) and in male participants (mean difference, −242.58 mm^3^; 95% CI, −428.60 to −56.55; *P* = .011).

**Interpretation:**

Individuals with chronic cough have a smaller volume of the anterior cingulate cortex, which is a brain region involved in cough suppression.

**Clinical Trial Registration:**

The Netherlands National Trial Registry (NTR; www.trialregister.nl) and the World Health Organization’s International Clinical Trials Registry Platform (ICTRP; www.who.int/ictrp/network/primary/en/) under the joint catalogue number NTR6831.

FOR EDITORIAL COMMENT, SEE PAGE 13

**Take-home Points**

**Study Question:** Are the volumes of the brain regions implicated in cough hypersensitivity different in adults with chronic cough compared with adults without chronic cough?

**Results:** Adults with chronic cough had smaller anterior cingulate cortex volume than adults without chronic cough. The volume difference in the anterior cingulate cortex was more pronounced in the left hemisphere and in men.

**Interpretation:** The observed volumetric alterations in the anterior cingulate cortex lends credence to the mechanistic relevance of impaired cough suppression in adults with chronic cough.

Chronic cough, defined as a cough that lasts more than 8 weeks, is one of the most common reasons for a primary care visit because it affects 2% to 18% of people worldwide.^1^ It is associated with significant physical and psychosocial complications, exacerbates underlying medical conditions,^2^, ^3^, ^4^ and imposes a huge clinical and financial burden on patients and health care systems.^5^^,^^6^ The management of chronic cough is challenging given that more than one-half of patients do not respond well to treatment (ie, refractory chronic cough)^7^ and that almost one-third of chronic cough cases remain unexplained despite a thorough diagnostic work up (ie, unexplained chronic cough).^5^ After several recommendations from clinical experts and scientific societies, which includes the European Respiratory Society, chronic cough has been recognized as a distinct clinical condition and recently was assigned the International Classification of Diseases, 10th revision, Clinical Modification code R05.3.^8^

The primary putative mechanism of chronic cough in adults, neural hypersensitivity,^9^ is sustained by neuroplastic changes, which are functional or structural maladaptation in the central and peripheral cough processing systems caused by repeated tussive stimuli exposure.^10^ Indeed, a study has shown that the density of airway epithelial sensory nerves is increased in chronic cough, which implies that peripheral sensory neuroplasticity plays a role in cough hypersensitivity.^11^ Similarly, several research groups have suggested that brain plasticity might contribute to central hyperexcitability and to inhibitory brain network dysfunction in adults with chronic cough.^12^^,^^13^

Experimental evidence of central sensitization in chronic cough has shown that when cough is evoked, brain activation patterns differ between healthy individuals and individuals with chronic cough.^13^ These functional changes in chronic cough may result in structural brain reorganization, which is detectable by MRI of the brain. In fact, structural brain changes that correlate with cough severity have been observed in patients with refractory chronic cough.^14^ Presently, evidence of (structural) brain plasticity in chronic cough is limited and mainly comes from clinical studies with small sample sizes.^13^^,^^14^ These studies have implicated some brain regions that are of interest to study further in a population-based sample. Furthermore, research into neuroplasticity in chronic cough could shed more light on the neuropathologic findings of different chronic cough phenotypes.

We proposed that specific brain regions that are known to be functionally distinct in individuals with cough hypersensitivity might also be structurally different in individuals with chronic cough compared with individuals without chronic cough. Therefore, we investigated structural brain alterations in chronic cough using brain MRI in adults from the Rotterdam Study, a large prospective population-based cohort study. Additionally, we examined whether regional brain volumes differed based on chronic cough phenotypes: namely explained and unexplained chronic cough.

## Study Design and Methods

### Study Setting and Study Population

The study population is composed of participants from the Rotterdam Study, a prospective population-based cohort study that enrolled 14,926 middle-aged and older adults (≥ 40 years old) who resided in the well-defined Ommoord district, a suburb of Rotterdam, the Netherlands. The design of the Rotterdam Study was described previously.^15^ Every 3 to 6 years, data are collected through home interviews and clinical examinations at the research center, in addition to data from medical records from general practitioners, hospitals, nursing homes, and pharmacies. Brain MRI has been included in the Rotterdam Study protocol since 2005, and participants are invited to repeat imaging every 3 to 4 years.^16^

**Figure 1:**
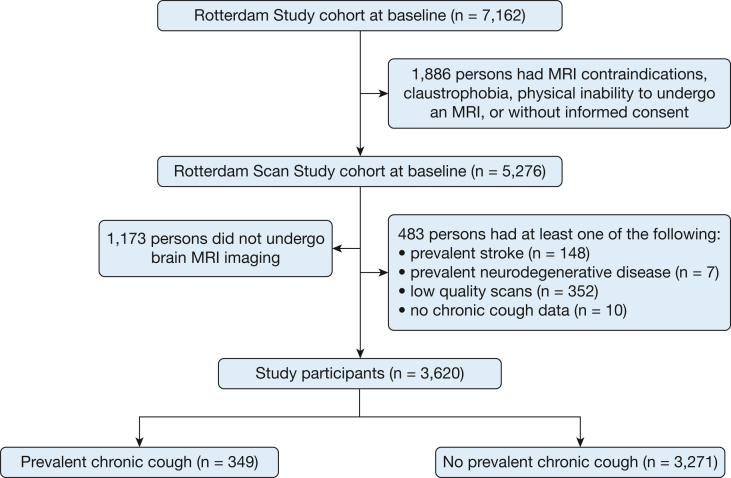
Strengthening the Reporting of Observational Studies in Epidemiology (STROBE) flow chart.

With the exception of scans with incomplete acquisition or scans with artifacts that prevent automated processing, MRI-defined cortical infarcts, or unreliable tissue segmentation, all MRI scans from the Rotterdam Study that were acquired between 2009 and 2014 were available for this study. Seventy-eight percent (n = 4,103) of the 5,276 adults in the Rotterdam Scan Study underwent brain MRI scanning after excluding adults with MRI contraindications, claustrophobia, physical inability to undergo an MRI, or without informed consent (n = 1,886). Chronic cough was assessed during the same period of investigation as the brain MRI imaging (from December 2008 to May 2014), and data were available in 99.7% (n = 7,141) of the Rotterdam Study cohort at baseline (n = 7,162). Participants with clinical diagnoses of stroke (n = 148) or neurodegenerative disease (dementia, Parkinsonism, or Parkinson disease) (n = 7), participants who did not complete the interview on chronic cough (n = 10), and participants with low-quality scans (n = 352) were also excluded because of their potential impact on brain volume. Moreover, participants with chronic cough (n = 48; 12.1%) had a higher proportion of poor-quality MRI scans than participants without chronic cough (n = 283; 8.0%; *P* = .005). Overall, this study included 3,620 participants with complete data for chronic cough and brain MRI who were free of clinically diagnosed stroke, dementia, Parkinsonism, or Parkinson disease (Fig 1). In terms of the assessment timeline, the majority of study participants (91.1%, n = 3,298) had chronic cough status assessed at least 8 weeks prior to brain MRI; 8.1% of participants (n = 293) had chronic cough assessed less than 8 weeks before brain MRI; and 0.8% of participants (n = 29) had chronic cough assessment after brain MRI (mean number of days between chronic cough assessment and brain MRI, 135; SD, 94 days).

### Assessment of Chronic Cough

In accordance with most epidemiologic studies, chronic cough was defined as daily coughing lasting for at least 3 months. The question “Did you cough almost every day for 3 consecutive months or more in the last 2 years?” was used to assess chronic cough.^17^ Participants who answered “no” were classified as having no chronic cough, whereas participants who answered “yes” were classified as having chronic cough. Furthermore, chronic cough was classified as “unexplained” if there was no evidence for the presence of known risk factors of chronic cough, in particular current smoking, use of angiotensin-converting enzyme inhibitors, gastroesophageal reflux disease (GERD), chronic rhinosinusitis, asthma, COPD, and lung cancer.^17^ Smoking status was assessed by interview, and pharmacy data were used to assess exposure to angiotensin-converting enzyme inhibitors (Anatomical Therapeutic Chemical code [ATC] C09A, C09B). Pharmacy data were used as a proxy to define GERD and chronic rhinosinusitis. Participants with GERD were defined as having received at least one prescription for acid-related disorders such as peptic ulcer or reflux disease (ATC A02B) prior to and within 2 years of baseline. Chronic rhinosinusitis was also defined as having received at least one prescription for nasal steroids (ATC R01AD) in the 2 years preceding baseline. Asthma was physician-diagnosed, and COPD cases were validated with the use of spirometry data and medical records. The Dutch cancer registry was used to identify lung cancer cases.

### Protocol for Brain MRI Acquisition and Image Processing

The imaging protocol and data processing method have been described in detail.^16^ To summarize, the brain MRI scanning was performed on the same single 1.5-T MRI scanner (GE Healthcare). Four noncontrast high-resolution axial sequences were acquired: a T1-weighted sequence (voxel size, 0.49 × 0.49 × 1.6 mm^3^), a proton density-weighted sequence (voxel size, 0.6 × 0.98 × 1.6 mm^3^), a fluid-attenuated inversion recovery sequence (voxel size, 0.78 × 1.12 × 2.5 mm^3^), and a T2^∗^-weighted gradient-recalled-echo sequence (voxel size, 0.78 × 1.12 × 1.6 mm^3^). To calculate total intracranial volume (ICV) and regional brain volumes of interest for each participant, brain MRI data were analyzed automatically with the use of the FreeSurfer parcellation (Desikan-Killiany Atlas) and segmentation software version 6.0 (http://surfer.nmr.mgh.harvard.edu/) that generates volumes in cubic millimeters. A standardized image analysis workflow was developed, validated, and used for all imaging data to enable the objective, precise, and reproducible extraction of brain images.^16^ Brain MRI with insufficient quality for analyses were excluded with the use of an automated tool that assesses artifacts related to motion.^18^^,^^19^

### Delineation of Brain Region of Interest

e-Table 1 provides an overview of previously published brain regions of interest that are implicated in chronic cough, which we focused on in the present study. The brain regions of interest were based on existing literature^13^^,^^14^ and include the anterior cingulate cortex, frontal pole, inferior frontal gyrus, insula cortex, middle frontal gyrus, prefrontal cortex, nucleus cuneiformis, and periaqueductal gray. For the current study, the brain regions of interest were derived from Freesurfer parcellations. The anterior cingulate volume was calculated by addition of the rostral and caudal anterior cingulate cortical volumes. The volume of the inferior frontal gyrus was calculated by the addition of the volumes of the pars opercularis, pars triangularis, and pars orbitalis. The middle frontal gyrus volume was calculated by the addition of the rostral and caudal middle frontal volumes. To estimate the volume of the prefrontal cortex, the volumes of the superior frontal gyrus, rostral middle frontal gyrus, caudal middle frontal gyrus, pars opercularis, pars triangularis, pars orbitalis, lateral orbitofrontal, medial orbitofrontal, and frontal pole were added together. FreeSurfer currently does not segment the nucleus cuneiformis or the periaqueductal gray; therefore, these brain structures were not assessed in this study.

### Assessment of Other Covariates

Participants’ age, sex, BMI (kg/m^2^), smoking status (never, former, current), and chronic pain were all assessed at baseline. A questionnaire (“Have you been in pain in the last 6 months?”) was used to assess chronic pain, and participants were instructed to select one of the following responses: “No,” “Yes, daily,” “Yes, weekly,” or “Yes, several times/monthly.” Participants were then grouped as either having no chronic pain or having chronic (daily/weekly/monthly) pain.^20^ The Dutch version of the Center for Epidemiologic Studies Depression (CESD) scale was used to assess depressive symptoms.^21^ The CESD scale, which ranges from 0 to 60, assesses the severity of self-reported depressive symptoms; higher scores indicate more severe symptoms. Clinically relevant depressive symptoms were defined as a score above 16.^21^

### Ethical Approval

The Rotterdam Study was approved by the Erasmus Medical Centre’s Medical Ethics Committee (registration number MEC 02.1015) and the Dutch Ministry of Health, Welfare, and Sport (Population Screening Act WBO, license number 1071272-159521-PG), and it was registered with the Netherlands National Trial Registry (www.trialregister.nl) and the World Health Organization’s International Clinical Trials Registry Platform (www.who.int/ictrp/network/primary/en/) under the joint catalogue number NTR6831. All participants provided written informed consent to participate in the study and to have their medical records collected.^15^

### Statistical Analysis

The demographic and clinical baseline characteristics of the study participants were presented with the use of descriptive statistics and compared based on chronic cough status. The *t*-test was used to compare normally distributed variables, which were presented as means with SDs. Mann-Whitney tests were performed for skewed continuous variables, and the median and interquartile range were reported. Chi-square test was used to compare categoric data that were presented as counts with percentages.

We investigated the association between chronic cough and predefined regional brain volumes using multivariable linear regression models that were adjusted for ICV, age, and sex (model 1) to account for sex- and age-differences in brain volumes. The independent variable was chronic cough (yes or no), and the dependent variables were predefined regional brain volumes. In model 2, we additionally adjusted for age^3^ (as cubic function, meaning polynomial function of degree 3), age and sex interaction, smoking, asthma, COPD, clinically relevant depressive symptoms (CESD score, > 16), and chronic pain, given that they are prevalent in chronic cough,^17^^,^^20^^,^^22^ and have been associated with volumetric brain changes in adults.^23^, ^24^, ^25^, ^26^, ^27^, ^28^ Furthermore, we estimated the annualized percentage volume decline rate, examined hemispheric lateralization, and performed stratified analyses for sex, focusing on brain regions that demonstrated statistical significance. We also explored the relationship between chronic cough phenotype (explained and unexplained) and regional brain volumes using multivariable linear regression models that were adjusted for potential confounding factors (models 1 and 2). Finally, to ensure a plausible temporal association between chronic cough and regional brain volumes of interest, we performed a sensitivity analysis (e-Table 2), excluding 29 participants whose chronic cough status was assessed after brain MRI and 293 participants whose chronic cough status was assessed less than 8 weeks before brain MRI; especially given that any observed brain volume difference in these participants, if any, might be unlikely due to the long-term effects of chronic cough. A probability value of < .05 was used to determine statistical significance. All statistical analyses were performed with the use of SPSS statistical software (version 28; IBM SPSS Statistics for Windows; IBM Corp).

## Results

### Characteristics of the Study Participants

The demographic and clinical characteristics of the study participants are presented in Table 1. At baseline, 9.6% (No. = 349) of the 3,620 participants (mean aged 68.5 ± 9.0 years; 54.6% female) reported chronic cough within 2 years before the interview. Current smoking (19.5% vs 10.7%; *P* < .001), chronic rhinosinusitis (17.5% vs 10.1%; *P* < .001), GERD (51.3% vs 39.1%; *P* < .001), asthma (14.3% vs 6.2%; *P* < .001), COPD (27.6% vs 13.9%; *P* < .001), lung cancer (1.4% vs 0.2%; *P* = .001), chronic pain (58.5% vs 51.2%; *P* < .001), and clinically relevant depressive symptoms (11.8% vs 7.2%; *P* < .001) were more prevalent among individuals with chronic cough compared with individuals without chronic cough. The mean ICV did not differ significantly according to chronic cough status (*P* = .708).

**Table 1:** Baseline Characteristics of the Study Population

Baseline Characteristics	Total (N = 3,620)	No Chronic Cough (n = 3,271)	Chronic Cough (n = 349)	*P* Value
Age, mean ± SD, y	68.5 ± 9.0	68.5 ± 9.0	68.4 ± 9.2	.426
Female sex, No. (%)	1,977 (54.6)	1,790 (54.7)	187 (53.6)	.684
BMI, median (interquartile range), kg/m^2^	26.7 (24.5 to 29.4)	26.7 (24.5 to 29.4)	26.9 (24.1 to 29.8)	.625
Smoking, No. (%)				< .001
Never	1,280 (35.4)	1,169 (35.7)	111 (31.8)	
Past	1,923 (53.1)	1,753 (53.6)	170 (48.7)	
Current	417 (11.5)	349 (10.7)	68 (19.5)	
Angiotensin-converting enzyme inhibitor use, No. (%)	527 (14.6)	470 (14.4)	57 (16.3)	.323
Baseline comorbidities, No. (%)				
Chronic rhinosinusitis	393 (10.9)	332 (10.1)	61 (17.5)	< .001
Gastroesophageal reflux disease	1,458 (40.3)	1,279 (39.1)	179 (51.3)	< .001
Asthma	253 (7.0)	203 (6.2)	50 (14.3)	< .001
COPD	504 (15.3)	415 (13.9)	89 (27.6)	< .001
Lung cancer	10 (0.3)	5 (0.2)	5 (1.4)	.001
Chronic pain	1,880 (51.9)	1,676 (51.2)	204 (58.5)	.010
Center for Epidemiological Studies Depression Scale score > 16	276 (7.7)	235 (7.2)	41 (11.8)	.002
Total intracranial volume, mean ± SD, 10^3^mm^3^	1,486 ± 157	1,486 ± 157	1,483 ± 162	.708

### Regional Brain Volumetric Differences According to Chronic Cough Status

The differences in regional brain volume based on chronic cough status are shown in Table 2. The age, sex, and ICV adjusted mean volumes of the anterior cingulate cortex (mean difference, −158.44 mm^3^; 95% CI, −271.64 to −45.23; *P* = .006) and middle frontal gyrus (mean difference, −365.70 mm^3^; 95% CI, −719.32 to −12.09; *P* = .043) were significantly smaller in participants with chronic cough compared with participants without chronic cough. Furthermore, the age, sex, and ICV adjusted mean volume of the following brain regions did not differ significantly by chronic cough status: frontal pole (mean difference, −4.41 mm^3^; 95% CI, −36.51 to 27.69; *P* = .788), inferior frontal gyrus (mean difference, −43.56 mm^3^; 95% CI, −237.48 to 150.36; *P* = .660), insula cortex (mean difference, −87.09 mm^3^; 95% CI, −204.54 to 30.36; *P* = .146), middle temporal gyrus (mean difference, −121.16 mm^3^; 95% CI, −323.58 to 81.27; *P* = .241), and prefrontal cortex (mean difference, −737.23 mm^3^; 95% CI, −1536.87 to 62.42; *P* = .071).

**Table 2:** Differences in Regional Brain Volume According to Chronic Cough Status

Region of Interest	Region of Interest Volume, Mean ± SD, mm^3^			Adjusted Mean Region of Interest Volume Difference, β (95% CI)			
	Total Sample	No Chronic Cough	Chronic Cough	Model 1^a^	*P* Value	Model 2^b^	*P* Value
Anterior cingulate cortex	7,505 ± 1,245	7,522 ± 1,254	7,348 ± 1,140	−58.44 (−271.64 to −45.23)	.006	−126.16 (−245.67 to −6.66)	.039
Frontal pole	2,105 ± 304	2,105 ± 302	2,100 ± 323	−4.41 (−36.51 to 27.69)	.788	−1.59 (−35.55 to 32.38)	.927
Inferior frontal gyrus	19,214 ± 2,255	19,220 ± 2,240	19,153 ± 2,386	−43.56 (−237.48 to 150.36)	.660	15.32 (−189.71 to 220.36)	.884
Insula cortex	13,546 ± 1,507	13,556 ± 1,514	13,450 ± 1,440	−87.09 (−204.54 to 30.36)	.146	−71.20 (−195.34 to 52.95)	.261
Middle frontal gyrus	39,336 ± 4,951	39,377 ± 4,946	38,943 ± 4,989	−365.70 (−719.32 to ˗12.09)	.043	−258.03 (−632.41 to 116.36)	.177
Middle temporal gyrus	20,108 ± 2736	20,122 ± 2,736	1,9975 ± 2,732	−121.16 (−323.58 to 81.27)	.241	−102.93 (−316.62 to 110.75)	.345
Prefrontal cortex	124,648 ± 12,998	124,734 ± 12,969	123,813 ± 13,254	−737.23 (−1,536.87 to 62.42)	.071	−354.69 (−1,195.04 to 485.67)	.408

a Adjusted for age, sex, and intracranial volume.

b Adjusted for age (cubic), sex, the interaction between age and sex, intracranial volume, smoking, asthma, COPD, Center for Epidemiological Studies Depression Scale score > 16, and chronic pain.

After adjustment for age (cubic), sex, the interaction between age and sex, smoking, asthma, COPD, CESD score > 16, chronic pain, and ICV, the lower brain volume associated with chronic cough remained significant only in the anterior cingulate cortex, with a mean volume difference of −1.7% (mean difference, −126.16 mm^3^; 95% CI, −245.67 to −6.66; *P* = .039). Given that the sex-adjusted annualized volume decline rate in the anterior cingulate cortex was −8.21 mm^3^ (95% CI, −12.44 to −3.99; *P* < .001), the adjusted mean volume difference in the anterior cingulate cortex associated with chronic cough (−126.16 mm^3^) represented a 15.4 year age difference.

**Figure 2:**
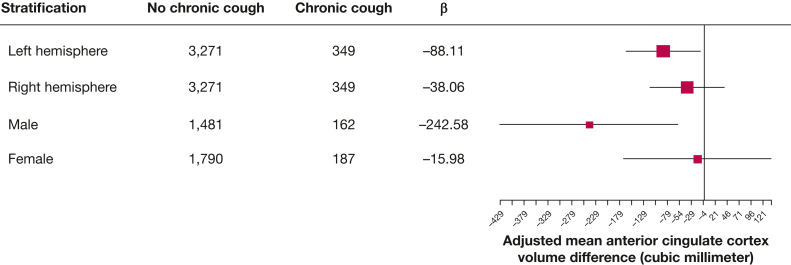
Difference in the anterior cingulate cortex volume of different strata according to chronic cough status.

In addition, the sensitivity analyses (Fig 2) show that the association of chronic cough with anterior cingulate cortex is more pronounced in the left hemisphere (mean difference, −88.11 mm^3^; 95% CI, −165.16 to −11.06; *P* = .025) than in the right (mean difference, −38.06 mm^3^; 95% CI, −115.64 to 39.53; *P* = .336), which indicates that participants with chronic cough had a 2.2% lower left anterior cingulate cortex volume than participants without chronic cough. The association was also stronger in male participants (mean difference, −242.58 mm^3^; 95% CI, −428.60 to −56.55; *P* = .011) compared with female participants (mean difference, −15.98 mm^3^; 95% CI, −169.32 to 137.35; *P* = .838), which indicates a 3.0% lower total anterior cingulate cortex volume in male participants with chronic cough.

Table 3 displays differences in regional brain volume according to chronic cough phenotype: unexplained chronic cough (n = 75; 2.1%) vs explained chronic cough (n = 274; 7.6%). Regional brain volumes of interest did not differ significantly between participants with unexplained chronic cough and participants without chronic cough. Likewise, participants with explained chronic cough and participants without chronic cough had comparable regional brain volumes, with the exception of the anterior cingulate cortex that showed a significant volume difference of −2.2% (mean difference, −67.50 mm^3^; 95% CI, −299.98 to −35.01; *P* = .013).

**Table 3:** Differences in Regional Brain Volume According to Chronic Cough Phenotype

Region of Interest	Region of Interest Volume, Mean ± SD, mm^3^			Adjusted Mean Region of Interest Volume Difference,^a^ β (95% CI)			
	No Chronic Cough	Unexplained Chronic Cough	Explained Chronic Cough	Unexplained Chronic Cough	*P* Value	Explained Chronic Cough	*P* Value
Anterior cingulate cortex	7,522 ± 1,254	7,380 ± 1,160	7,327 ± 1,133	−51.48 (−303.44 to 200.48)	.689	−167.50 (−299.98 to −35.01)	.013
Frontal pole	2,105 ± 302	2,103 ± 337	2,100 ± 321	6.29 (−65.35 to 77.93)	.863	−4.24 (−41.90 to 33.43)	.826
Inferior frontal gyrus	19,220 ± 2,240	19,150 ± 1,939	19,136 ± 2,480	19.41 (−413.02 to 451.83)	.930	−10.91 (−238.28 to 216.47)	.925
Insula cortex	13,556 ± 1,514	13,381 ± 1,586	13,442 ± 1,410	−111.09 (−372.87 to 150.70)	.405	−88.27 (−225.92 to 49.38)	.209
Middle frontal gyrus	39,377 ± 4,946	39,098 ± 4,773	38,858 ± 5,047	114.22 (−675.11 to 903.54)	.777	−414.40 (−829.40 to 0.63)	.050
Middle temporal gyrus	20,122 ± 2,736	19,985 ± 3,037	19,970 ± 2,641	−66.86 (−517.53 to 383.81)	.771	−108.47 (−345.44 to 128.50)	.370
Prefrontal cortex	124,734 ± 12,969	124,385 ± 12,648	123,560 ± 13,359	668.19 (−1,103.51 to 2,439.89)	.460	−738.86 (−1,670.43 to 192.72)	.120

a Model 2 was adjusted for age (cubic), sex, age∗sex, intracranial volume, smoking, asthma, COPD, Center for Epidemiological Studies Depression Scale score > 16, and chronic pain.

## Discussion

In this study, we investigated the volumes of seven brain regions that have been shown to be altered functionally in chronic cough. We found that the volumes of the frontal pole, inferior frontal, insula, middle frontal, middle temporal, and prefrontal cortices did not differ significantly between participants with and without chronic cough. On the other hand, chronic cough was associated significantly with a smaller volume of the anterior cingulate cortex, independent of ICV, age, sex, smoking, asthma, COPD, depressive symptoms, and chronic pain. In addition, the negative correlation between chronic cough and the volume of the anterior cingulate cortex was more pronounced in male participants and in the left hemisphere.

Unlike clinical studies that observed functional differences in multiple brain regions in patients with refractory chronic cough whose condition had been well phenotyped,^13^^,^^14^ we could demonstrate only volumetric differences related to cough in a single brain region (the anterior cingulate cortex). There are several plausible explanations for this discrepancy. First, our study was population based and focused on structural differences associated with chronic cough, which could be a less sensitive marker than brain function. Moreover, although cortical activations in the studied brain regions are known to be distinct functionally in patients with chronic cough, their potential impact on brain structure may vary because of the degree of heterogeneity in chronic cough endotypes, the multiplicity of underlying clinical conditions, and the diversity of triggering factors, including smoking and postviral cough, among others.^3^^,^^17^ Nonetheless, our findings are consistent with previous research on differences in brain volume in chronic cough. Namgung et al^14^ recently found that patients with refractory chronic cough have a lower gray matter volume in the brain region comprising the middle frontal gyrus than patients in the control group, independent of age and sex. Similarly, we observed that, independent of ICV, age, and sex, participants with chronic cough had smaller middle frontal volume compared with participants without chronic cough. However, this association was no longer statistically significant after further adjustment for smoking, asthma, COPD, depressive symptoms, and chronic pain.

Previous studies in patients with refractory chronic cough have demonstrated reduced brain activation in the anterior cingulate cortex, which is a brain region implicated in dysfunctional cough suppression.^13^^,^^29^ Indeed, a functional brain imaging study by Ando et al^13^ found that blood oxygen level-dependent signals in the anterior midcingulate cortex were lower in patients with cough hypersensitivity compared with healthy subjects after a capsaicin inhalation challenge. In contrast to patients with chronic cough, experimental studies have shown that, in healthy, cough-free subjects, the intensity of the urge to cough positively correlates with anterior cingulate cortical activation, which indicates a preserved compensatory physiologic response to cough stimuli.^30^^,^^31^ Perhaps, cough inhibitory control may be impaired in chronic cough because of maladaptive structural changes in the anterior cingulate cortex, which may act as an intermediary for central sensitization, resulting in cough hypersensitivity. Additionally, the anterior cingulate cortex is involved in the cognitive and emotional processing of sensory signals,^32^ and structural changes in this region may have a negative impact on emotional cough processing and perceived cough severity in patients with chronic cough who are predisposed to psychomorbidities, such as depressive symptoms and recurrent depression.^22^ It is plausible that these pathologic changes contribute to a switch from sensory to emotional circuits in chronic cough state. For example, in chronic pain, a condition with a similar neurobiologic mechanism and therapeutic target as chronic cough,^20^ the anterior cingulate cortex is reduced volumetrically and modulates pain-related negative emotion.^33^

We found that chronic cough-related morphometric alterations in the anterior cingulate cortex were driven mainly by people with risk factors or treatable chronic cough traits, which indicates that this group may have more extensive structural brain changes. Although we do not have a direct explanation for this finding, most medical conditions associated with chronic cough, such as chronic obstructive airway disease, are chronic in nature and are modifiable chronic cough risk factors, such as smoking, are habitual.^17^ Thus, patients with chronic cough who are exposed to these risk factors may experience persistent noxious input sufficient to initiate and sustain central neuroplastic changes that promote cough hypersensitivity.

Atrophic changes in the brain are known to increase with age.^34^ Importantly, age-related brain volume decline is greater in men^35^ and has a predilection, among others, for the anterior cingulate cortex.^36^ The present study had a high proportion of older participants, and found that the association between chronic cough and a smaller anterior cingulate cortex volume was stronger in male participants than in female participants. It is unclear therefore how much of the observed differences in the volume of the anterior cingulate cortex are due to aging as opposed to the possible structural maladaptive effects of chronic cough in a normally aging brain. Nonetheless, we corrected for age and the interaction between age and sex in our study and found that the participants with and without chronic cough had comparable age distributions, so the residual confounding of age in our findings is expected to be minimal. Furthermore, although the absolute difference in anterior cingulate cortex volume associated with chronic cough was small in our study population, the anterior cingulate cortex volume decline rate per year increase in age was -8.21 mm^3^, whereas the volume difference in the anterior cingulate cortex associated with chronic cough was -126.16 mm^3^, which indicates a clinically significant volume difference equivalent to more than a decade of age difference in participants with chronic cough.

To the best of our knowledge, this is the first large population-based observational study to use brain MRI to investigate differences in structural brain volumes in adults with chronic cough. Our research has several strengths. First, we focused on the specific brain regions previously linked to dysfunctional brain activity in chronic cough and excluded people who had prevalent stroke or neurodegenerative disease, thereby limiting spurious findings. Second, we accounted for intersex and age differences in brain volumes and adjusted for other relevant confounding factors. However, we assessed only chronic cough in the 2 years preceding the baseline. Perhaps, people who already had chronic cough earlier in life might have a “duration-dependent” effect on brain volume. Also, participants with chronic cough had a higher proportion of low-quality MRI scan than participants without chronic cough, which indicates a group-related bias. Furthermore, we did not have data on chronic rhinosinusitis and GERD; hence, we used medication for these indications as a proxy to identify participants with these medical conditions. This may have resulted in disease misclassification, because subjects with fewer or no prescriptions may have been underdiagnosed. Last, the cross-sectional design of our study limits the inference of a causal relationship between chronic cough and the observed volume differences in the anterior cingulate cortex. Therefore, a longitudinal study of brain volumetric changes in chronic cough is required to confirm our findings. Overall, our study contributes to our understanding of the nature of neuropathologic changes in chronic cough and provides epidemiologic evidence of possible central neuroplasticity in chronic cough. Nevertheless, more research is needed to understand the implications of our findings in pharmacologic and nonpharmacologic interventions for chronic cough in adults.

## Interpretation

In summary, we observed that chronic cough is associated with a smaller anterior cingulate cortex volume, which lends credence to the potential role of structural brain changes in cough hypersensitivity. Interestingly, the differences in brain volume that are associated with chronic cough were confined to a region previously implicated in dysfunctional cough suppression and emotional cough processing, which indicates that impaired cough control and cough input amplification are central mechanisms of chronic cough in adults. Our findings may shed light on potential therapeutic targets for adults with chronic cough.

## Funding/Support

This study was supported in part by a research grant from the Investigator-Initiated Studies (ISS) Program of Merck Sharp & Dohme (MSD, Belgium; A19TT2089).

## Financial/Nonfinancial Disclosures

None declared.

## Supplementary Data

e-Online Data
