# Supplementary material for: Chronic Cough-Related Differences in Brain Morphometry in Adults: A Population-Based Study
Source: Chest. 2023 Feb 11;164(1):169–78. doi: 10.1016/j.chest.2023.02.007 (PMC10329265; doi:10.1016/j.chest.2023.02.007)
Supplement: e-Online Data [file mmc1.docx]

**Supplemental Table 1. Brain regions with differential activation or structural changes in chronic cough when compared to healthy subjects as described in the literature.**

| **No.** | **Brain Region** | **Brain activity (+ /-) & volumetric change** | **Sample size** | **FreeSurfer parcellate equivalent** |
| --- | --- | --- | --- | --- |
| 1. | Anterior cingulate cortex ^13^ | - | 32 | lh_caudalanteriorcingulate, rh_caudalanteriorcingulate  lh_rostralanteriorcingulate, rh_rostralanteriorcingulate |
| 2. | Frontal pole ^14^ | Smaller volume | 30 | lh_frontalpole, rh_frontalpole |
| 3. | Inferior frontal gyrus ^13^ | + | 32 | lh_parsopercularis, rh_parsopercularis  lh_parsorbitalis, rh_parsorbitalis,  lh_parstriangularis, rh_parstriangularis |
| 4. | Insula cortex ^13^ | + | 32 | lh_insula, rh_insula |
| 5. | Middle frontal gyrus ^14^ | Smaller volume | 30 | lh_caudalmiddlefrontal, rh_caudalmiddlefrontal  lh_rostralmiddlefrontal, rh_rostralmiddlefrontal |
| 6. | Middle temporal gyrus ^14^ | + | 30 | lh_middletemporal, rh_middletemporal |
| 7. | Prefrontal cortex ^13^ | - | 32 | Superior frontal: lh_superiorfrontal, rh_superiorfrontal  Orbitofrontal: lh_lateralorbitofrontal, rh_lateralorbitofrontal,  lh_medialorbitofrontal, rh_medialorbitofrontal.  Inferior frontal, Middle frontal, Frontal pole |
| 8. | Nucleus cuneiformis ^13^ | + | 32 | Not available |
| 9. | Periaqueductal gray ^13^ | + | 32 | Not available |

**Supplemental Table 2. Differences in regional brain volume according to chronic cough status: Sensitivity analyses in participants had chronic cough status assessed at least 8 weeks prior to brain MR imaging (n=3,298).**

| **ROI** | **#Adjusted Mean ROI volume difference (mm^3^) (β (95% CI))** | | | | |
| --- | --- | --- | --- | --- | --- |
|  | **Main analyses (n=3,620)** | **p-value** | **Sensitivity analyses (n=3,298)** | **p-value** | |
| Anterior cingulate | –126.16 (–245.67, –6.66) | 0.039 | –128.94 (–254.00, –3.87) | | 0.043 |
| Frontal pole | –1.59 (–35.55, 32.38) | 0.927 | 4.11 (–31.26, 39.49) | | 0.820 |
| Inferior frontal | 15.32 (–189.71, 220.36) | 0.884 | –3.02 (–220.57, 214.53) | | 0.978 |
| Insula | –71.20 (–195.34, 52.95) | 0.261 | –72.51 (–201.71, 56.68) | | 0.271 |
| Middle frontal | –258.03 (–632.41, 116.36) | 0.177 | –193.30 (–590.39, 203.78) | | 0.340 |
| Middle temporal | –102.93 (–316.62, 110.75) | 0.345 | –74.19 (–299.95, 151.58) | | 0.519 |
| Prefrontal | –354.69 (–1195.04, 485.67) | 0.408 | –346.22 (–1237.15, 544.71) | | 0.446 |
| ROI – Brain Region of Interest. # – Adjusted for age, sex, and ICV. Model 2 – Adjusted for age (cubic), sex, age*sex, ICV, smoking, asthma, COPD, CESD score >16, and chronic pain.  Comment: the results of the sensitivity analysis were similar to those of the main analyses. | | | | | |
